# Metabolomic Analysis of Hexagenid Mayflies Exposed to Sublethal Concentrations of Naphthenic Acid

**DOI:** 10.3389/fmolb.2021.669082

**Published:** 2021-06-15

**Authors:** Sarah M. Pomfret, Robert B. Brua, Danielle Milani, Adam G. Yates

**Affiliations:** ^1^ StrEAMS Laboratory, Department of Geography and Canadian Rivers Institute, Western University, London, ON, Canada; ^2^ Watershed Hydrology and Ecology Research Division, Environment and Climate Change Canada, Saskatoon, SK, Canada; ^3^ Watershed Hydrology and Ecology Research Division, Environment and Climate Change Canada, Burlington, ON, Canada

**Keywords:** *Hexagenia*, oil sands process waters, microcosm experiment, sublethal effects, toxicity test, survival, growth, metabolome

## Abstract

The oil sands region in northeastern Alberta, Canada contain approximately 165 billion barrels of oil making it the third largest oil reserves in the world. However, processing of extracted bitumen generates vast amounts of toxic byproduct known as oil sands process waters. Naphthenic acids and associated sodium naphthenate salts are considered the primary toxic component of oil sands process waters. Although a significant body of work has been conducted on naphthenic acid toxicity at levels comparable to what is observed in current oil sands process waters, it is also important to understand any impacts of exposure to sublethal concentrations. We conducted a microcosm study using the mayfly *Hexagenia* spp*.* to identify sublethal impacts of naphthenic acid exposure on the survival, growth, and metabolome across a concentration gradient (0–100 μg L^−1^) of sodium naphthenate. Nuclear magnetic resonance-based metabolomic analyses were completed on both the polar and lipophilic extracted fractions of whole organism tissue. We observed a positive relationship between sodium naphthenate concentration and mean principal component score of the first axis of the polar metabolome indicating a shift in the metabolome with increasing naphthenic acid exposure. Eleven metabolites correlated with increased naphthenic acid concentration and included those involved in energy metabolism and apoptosis regulation. Survival and growth were both high and did not differ among concentrations, with the exception of a slight increase in mortality observed at the highest concentration. Although lethal concentrations of naphthenic acids in other studies are higher (150–56,200 μg L^−1^), our findings suggest that physiological changes in aquatic invertebrates may begin at substantially lower concentrations. These results have important implications for the release of naphthenic acids into surface waters in the Alberta oil sands region as an addition of even small volumes of oil sands process waters could initiate chronic effects in aquatic organisms. Results of this research will assist in the determination of appropriate discharge thresholds should oil sands process waters be considered for environmental release.

## Introduction

Oil production in Western Canada includes one of the largest bitumen extraction operations in the world. Bitumen is a dense, viscous mixture of heavy oil and sand that when processed produces large volumes of oil sands process water (OSPW), which generally amasses a volume up to four times greater than that of subsequent refined oil products (Holowenko et al., 2002). The Alberta government currently prohibits release of OSPW to aquatic environments (Giesy et al., 2010). One implication of this policy is the amassment of large volumes of OSPW in tailings ponds with no definitive plan for disposal or remediation (van den Heuvel, 2015). Although OSPW is not currently discharged to the aquatic environment, future policy changes, accidental releases, and seepage into natural waters all can pose a risk to aquatic environments if environmental outcomes are not well understood.

Naphthenic acids are a natural component of bitumen. However, naphthenic acids become concentrated in OSPW through the continual recycling and reuse of water during bitumen processing. There is general agreement that naphthenic acids are the primary contributor to toxicity observed in OSPW (Brown and Ulrich, 2015; Li et al., 2017). Concentrations of naphthenic acids in OSPW have been reported upwards of 120 mg L^−1^ (Holowenko et al., 2002). However, in the surrounding aquatic environments of the oil sands region, naphthenic acids are typically present in concentrations below 1,000 μg L^−1^ (Headley and McMartin, 2004; Kilgour et al., 2019). Indeed, Ross et al. (2012) suggested concentrations ranging from as low as less than 2 μg L^−1^ in the Athabasca River to 173 μg L^−1^ in sediment pore water. Similarly, Arens et al. (2017) provide a characterization of the naphthenic acid concentration in the Athabasca River region, reporting naphthenate ion concentrations in the Athabasca mainstem and tributaries between 0.66–51.62 μg L^−1^. Although intentional release to aquatic environments is currently prohibited, several research initiatives are underway to reduce the naphthenic acid attributed toxicity of OSPW with the goal of eventual release into aquatic ecosystems. However, even in the case that OSPW is never intentionally released into aquatic ecosystems, it is important to understand the ecological consequences of unintentional release.

Toxicity of OSPW, as a complex mixture containing naphthenic acid, has been well studied in several aquatic organisms, including *Daphnia*, chironomids, and several fish species (Li et al., 2017). Naphthenic acids have been demonstrated to result in a number of lethal and sublethal effects for various vertebrate and invertebrate species, even at relatively low concentrations when compared to raw OSPW. For example, Loughery et al. (2019) reported changes in the transcriptome of fish larvae at 1,250 μg L^−1^, and Bartlett et al. (2017) reported invertebrate LC_50_ values as low as 882 μg L^−1^. Sublethal impacts reported from studies examining naphthenic acid exposure in aquatic species have varied with reports of endocrine disruption (Kavanagh et al., 2012; Scarlett et al., 2012; Wang et al., 2015), increased oxidative stress (Marentette et al., 2017; Morandi et al., 2017), liver toxicity (Nero et al., 2006), and disruption of energy pathways (Melvin et al., 2013) at concentrations between 50–500 μg L^−1^. However, it is important to fully understand the impacts of naphthenic acids at currently observed environmental concentrations, as well as those concentrations potentially relevant in the future. Understanding the lowest concentrations at which naphthenic acids have the potential to generate adverse biological effects will aid in understanding ecosystem wide consequences and appropriate policy actions. Indeed, biological systems are highly interconnected and significant impacts at the community and ecosystem levels can be generated from even minor impacts to fitness outcomes of individual species (Kramer et al., 2011). It is therefore paramount that sublethal concentration thresholds at which aquatic species begin to be impacted by naphthenic acid toxicity be established in order to fully elucidate potential environmental outcomes.

Metabolomics could be a useful tool in elucidating thresholds at which naphthenic acids initiate biological effects that may be precursory to observable population and community level impacts (Pomfret et al., 2020). Indeed, metabolomics has shown efficacy in determination of sublethal contaminant effects in various contexts and may be suitable for assessment of environmental effects of naphthenic acids (Viant et al., 2003). Changes in the metabolome are precursory to observable changes in fitness, and therefore, are likely to represent the first measurable outcome in relation to environmental stress. Moreover, metabolomics has been effectively applied to detect stressor effects in various species in environmental situations (Cappello et al., 2013; Davis et al., 2013; Skelton et al., 2014; Izral et al., 2021). It is thus likely that changes in the metabolome will occur at stressor concentrations lower than those of observable fitness outcomes, although metabolomic changes could also be predictive of reduced fitness outcomes on a longer, potentially even multigenerational, time scale.

The mayfly, *Hexagenia* spp., is ideal for monitoring effects of bitumen extractions using metabolomics due to the prevalence of this taxon in ecosystems across North America, including the Athabasca oil sands region (Clifford and Boerger, 1974). *Hexagenia* are organisms of interest in community level responses due to their position near the base of the food web as consumers of detritus. Therefore, the removal of this group of organisms has potential implications at a community level as they are often a significant food source for many aquatic and terrestrial species. Moreover, this group of mayflies are burrowers that consume detritus and naphthenic acids have been previously shown to bind to organic matter in sediment (Janfada et al., 2006), and thus this taxon’s habitat is where toxic effects may be strongest. *Hexagenia* also exhibit a fast growth rate and short maturation period, meaning that changes to growth and fecundity as a result of naphthenic acid contamination are likely to appear quickly within the population.

The goal of this study was to evaluate the effect of naphthenic acid exposure on growth, survival, and the metabolome of *Hexagenia* spp. nymphs. More specifically, we evaluated the growth, survival, and metabolome change across the tested concentration gradient to identify where impacts of naphthenic acid exposure begin to be measurable. To accomplish this, we used a controlled microcosm experiment to expose *Hexagenia* mayflies to a gradient of naphthenic acid concentrations representing their current range of conditions reported in aquatic ecosystems in the surface mining region of the Alberta oil sands. Understanding the impacts on the survival, growth, and change in metabolome of *Hexagenia* caused by exposure to naphthenic acid concentrations currently observed or expected in various environments will provide valuable information for higher order ecosystem predictions. In addition to closing a knowledge gap in relation to naphthenic acid toxicity, results of this study will be crucial to inform policy regarding potential future environmental management of OSPW.

## Materials and Methods

### Culture of *Hexagenia*

*Hexagenia* spp. nymphs used in our experiment were cultured from eggs collected in the evening from sexually mature females in June 2016 at the outflow of Lake St. Clair (42°20′22.1″N 82°55′51.6″W), near Windsor, Ontario, Canada. *Hexagenia* were cultured at the Canada Centre for Inland Waters in Burlington, Ontario, Canada. Culture tanks were washed with Extran®, 20% HCl, and thoroughly rinsed prior to use. Culture sediment (7.3% TOC) was collected in June 2016 from Long Point Marsh (42°35′21.30″N, −80°27′13.00 W) and stored at 4°C. Prior to use, sediment was filtered through a 250 µm sieve to homogenize sediment and remove any extraneous biological material. Each culture tank was lined with approximately 2 cm of sediment, filled with culture water (charcoal filtered, dechlorinated Burlington, Ontario, city water sourced from Lake Ontario), and warmed to 24°C. Twenty milliliters of food mixture (4 g ground Tetramin™, 3 g yeast, and 3 g cereal grass in 100 ml distilled water) was added to each tank 24 h prior to animal addition. Three hundred hatched individuals were placed in each tank and allowed to settle, prior to gentle aeration 1 h later. Cultures were fed weekly, each culture tank receiving approximately 0.8 g of food mixture every 7 days. Individual *Hexagenia* were 6–8 weeks of age at experiment start. Age and size of individuals were randomized among treatments.

### Sodium Naphthenate Characterization

**FIGURE 1:**
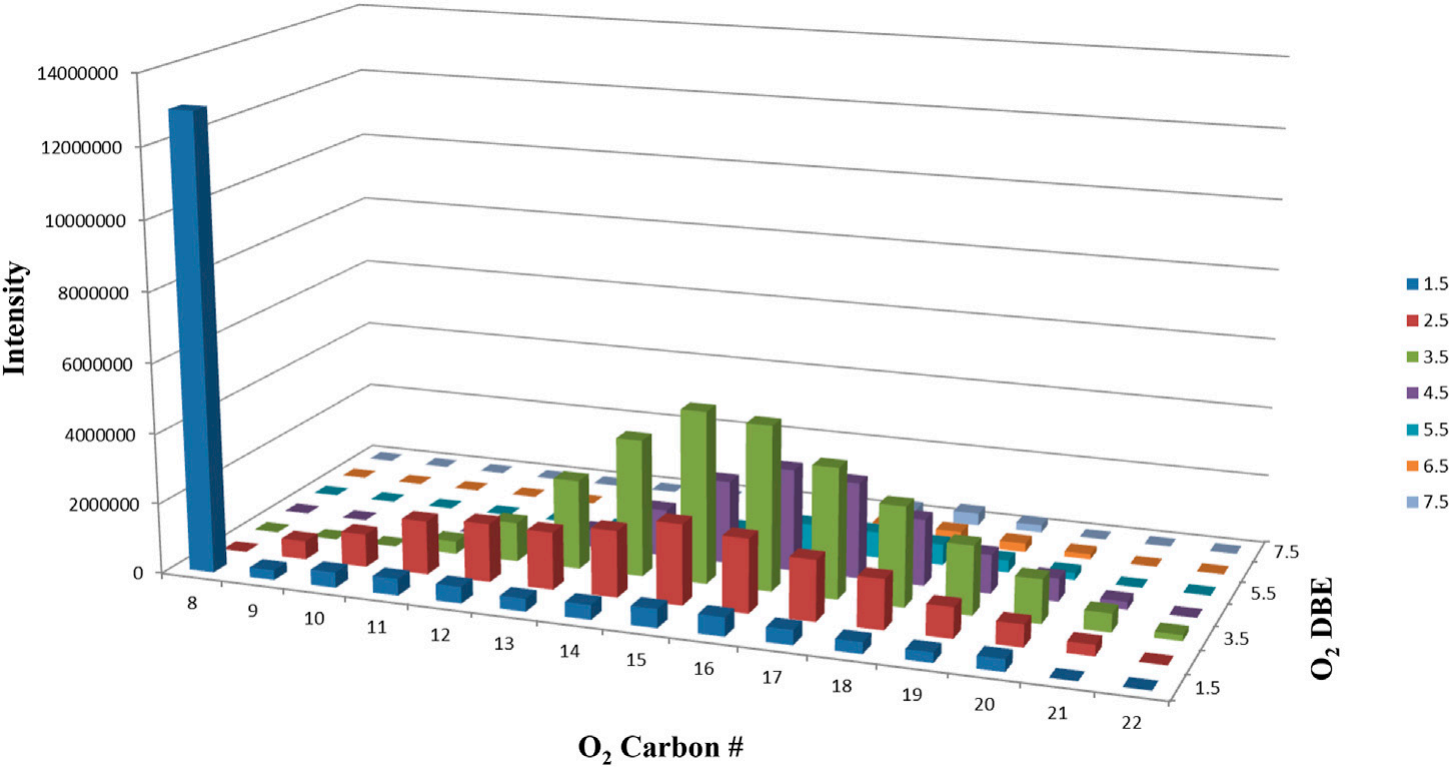
Structural distribution of O_2_ naphthenate species arranged by total carbon number and double bond equivalents (DBE) in a standard sodium naphthenate sample used in *Hexagenia* spp. toxicity testing.

Sodium naphthenate, the sodium salt of naphthenic acid, was purchased from TCI Chemicals America (Portland, Oregon, United States) and characterized by mass spectrometry. Analyses found the purchased naphthenate mixture to contain 99.8% O_2_ species, with the remaining 0.2% comprised of S_5_ species. OSPW derived naphthenic acid mixtures have been previously characterized to contain 40–86% O_2_ naphthenic acids (Marentette et al., 2015), which have been identified as the major toxic component of OSPW (Hughes et al., 2017). The sample ranged in carbon number from 8 to 22 with 1.5–7.5 double bond equivalents (Figure 1).

Experimental concentrations of sodium naphthenate were generated by serial dilution. Sodium naphthenate (Na–NA) was first dissolved to create a stock solution of verified concentration (55,730 ± 5.18 (SD) µg Na–NA L^−1^). Stock solution was added to microcosm jars to achieve desired concentrations (0, 0.0001, 0.001, 0.006, 0.01, 0.04, 0.08, 0.1, 0.4, 0.8, 1, 6, 10, 60, and 100 μg L^−1^). Concentrations were chosen based on preliminary data of acute sodium naphthenate toxicity to mayflies prior to publication by (Howland et al., 2019a).

### Experimental Procedures

Our study used microcosms (i.e., small scale simulated environments) to evaluate survival, growth, and biochemical responses of *Hexagenia* spp. to naphthenic acids. Microcosms are ideal for use in situations where environmental control and experimental repeatability are required while maintaining specific environmental conditions (Pirrone et al., 2018). Moreover, the use of microcosms is well established for toxicity testing, owing primarily to the increased transferability of results between field and laboratory environments as microcosms can closely mimic those of target ecosystems (Alexander et al., 2014).

Prior to the experiment, all microcosms (2 L glass jars) were washed with Extran® and 20% HCl. Sediment used in the exposures was a mixture of culture sediment (Long Point Marsh) and sediment collected from Long Point Bay (42°36′0″N, 80°13′60″W). Each sediment was sieved through a 250-µm sieve and then mixed in a 2:3 ratio by volume of marsh and bay sediment to achieve a total organic carbon content of 2%, similar to the value found in the Athabasca River (R.B. Brua, unpublished data). Sediment (250 ml) was added to each microcosm followed by the addition of 1.2 L culture water. Sediment was allowed to settle in the microcosms for 24 h before the addition of specified volumes of sodium naphthenate stock solution. Four microcosms were assigned to each treatment concentration (*n* = 60 microcosms) with 18 mayflies randomly assigned to each microcosm (*n* = 1,080). After sodium naphthenate addition, microcosms were aerated to >90% dissolved oxygen saturation for 6 days prior to experiment start. After the 6-day period, aeration was stopped to allow addition of 18 mayflies and then restarted 1 h after addition. Individual mayflies were massed in groups of nine to allow for growth determinations prior to placement in the randomly assigned microcosm.

Microcosms were placed in a temperature-controlled chamber at 24°C for the duration of the 21-days exposure period. Each microcosm was aerated consistently to ensure adequate dissolved oxygen, and exposed to a 16:8 h light:dark cycle. Microcosms also received 50 mg of culture food mixture once weekly to prevent starvation. Temperature, specific conductance (µS cm^−1^), pH, and percent dissolved oxygen was monitored weekly (4 times in total) in each microcosm using a YSI ProPlus multimeter.

### Survival and Growth

*Hexagenia* spp. were counted and massed in groups of nine corresponding to their assigned microcosm at the beginning of the experiment. The total mass (wet weight) of the group in each microcosm was divided by the number of individuals to determine the initial average mass of each individual. At the conclusion of the exposure period, individual mayflies were separated from exposure sediment and the surviving mayflies were enumerated. If fewer than 18 mayflies survived in a microcosm, the group was split as evenly as possible and mayflies were massed in groups of nine, eight, or seven to determine the average growth of all surviving individuals in each microcosm. Each group was placed in a cryotube, flash frozen in liquid nitrogen and stored at -80°C until tissue extraction.

### Metabolite Extraction

Groups of 7–9 mayflies were pooled for extraction of metabolites from tissues to obtain suitable mass for nuclear magnetic resonance (NMR) analysis. Two pooled groups of mayflies were made from each microcosm, creating a sample size of 8 pooled samples for metabolomic analyses per sodium naphthenate concentration. Mayflies were lyophilized overnight and subsequently homogenized using a Precellys homogenizer. Ten mg (dry weight) of ground tissue was added to a 2 ml Eppendorf tube. Metabolites were extracted using a 2:2:1.8 chloroform:methanol:water extraction (Viant et al., 2003). Briefly, ice-cold methanol (0.6 ml) and ice-cold Millipore water (0.27 ml) were added to each sample and then vortexed three times for 15 s, followed by centrifugation at 13,400 rpm for 10 min. The supernatant was then collected and transferred to a new Eppendorf vial, where ice-cold chloroform (0.6 ml) and ice-cold Millipore water (0.27 ml) was added. Samples were vortexed for 60 s, allowed to partition on ice for 10 min, and centrifuged at 13,400 rpm for 10 min. The methanol (upper) and chloroform (lower) layers were then removed and placed into separate Eppendorf® tubes. Solvents were then evaporated using a Speedvac Evapoconcentrator (ThermoScientifc), and subsequently stored at −80°C until resuspension in appropriate solvent (sodium phosphate buffer for polar metabolites and 2:1 deuterated chloroform:methanol mixture for lipophilic metabolites) for NMR analyses.

### NMR Spectroscopy

NMR analyses were completed on both the polar and lipophilic fraction of the metabolome. All ^1^H 1D NMR spectra were acquired at 298 K using a Bruker Avance 600 MHz spectrometer equipped with a 5 mm TXI broadband probe and operating at 600.17 MHz. The instrument was locked to the appropriate deuterium resonance (D_2_O for polar metabolites, Methanol-d4 for lipophilic metabolites), tuned, and shimmed prior to each spectral acquisition.

Polar metabolites were resuspended in 0.75 ml 100 mM sodium phosphate buffer (pH = 7.0) in 90% H_2_O and 10% D_2_O with 3 mM NaN_3_ added as a preservative and 1 mM Trimethylsilylpropanoic acid (TMSP) as an internal standard. Samples were vortexed for 60 s, centrifuged for 5 min, and 0.6 ml of resuspended solution was then placed immediately in a 5 mm NMR tube for analysis. An excitation sculpting pulse sequence (Hwang and Shaka, 1995) with a 60° pulse was used to maximize water suppression. Data were acquired with a relaxation delay of 2 s, spectral width of 9,615.385 Hz, and 128 scans. Data were then Fourier transformed, manually phased, baseline corrected using a polynomial baseline correction, and referenced to TMSP. Spectra were processed with LB = 0.3 and zero filled to 32,768 data points.

Lipophilic metabolites were resuspended in 0.75 ml 2:1 chloroform-d:methanol-d_4_ mixture containing 0.5 mM tetramethylsilane (TMS) as an internal standard. Samples were vortexed for 60 s, centrifuged for 5 min, and 0.6 ml of resuspended solution was then placed immediately in a 5 mm NMR tube for analysis. A standard 90° pulse acquire sequence with a 2.7 s acquisition time and 3 s relaxation delay, 128 scans, and 12,019.230 Hz spectral width was used. Data were then Fourier transformed, manually phased, baseline corrected using a polynomial baseline correction, and referenced to TMS. Spectra were processed with LB = 0.3 and zero filled to 65,536 data points. All polar and lipid spectra were integrated and normalized to the area of the standard peak (TMSP or TMS) (Viant et al., 2003).

### Statistical Analysis

Statistical analysis on the growth, survival, and metabolomes was completed with Systat and MetaboAnalyst software. Mean growth and survival data were analyzed in Systat using a regression model to assess trends in growth and survival along the treatment gradient (*α* = 0.05). Growth and survival data are reported as mean **±** standard deviation.

Spectral data were exported to *Prometab* within MATLAB (The MathWorks, Natick, MA, United States) and binned from 0.8 to 10 ppm in 0.005 ppm wide bins and transformed with a general log transformation (Parsons et al., 2007) based on replicate measurements from several control metabolomes (6 replicates for the lipid metabolome, 5 replicates for the polar metabolome). Following binning, data sets were truncated to remove areas of noise and subsequently polar and lipophilic samples were analyzed via a principal component analysis (PCA) by MetaboAnalyst 4.0 (Chong et al., 2019). Interpretable principal components were selected based on the generated scree plots leading to the retention of the first two components for both the polar and lipophilic metabolomes. Mean PCA scores along principal component 1 and 2 were then analyzed using linear regression to evaluate associations with sodium naphthenate concentrations. Significant (*p* ≤ 0.05) regression models were further analyzed using PatternHunter in MetaboAnalyst. The PatternHunter feature searches the binned metabolite data for bins that have significant correlations (Pearson r), using false discovery rate (FDR) correction (*α* = 0.10), with the gradient of sodium naphthenate concentrations. Chenomx NMR suite 8.3 (Chenomx Inc.) and previously published metabolite chemical shift values (Fan, 1996) were used to identify metabolites within the significant (*p* ≤ 0.10) bins identified through the PatternHunter correlation analysis. The less conservative alpha of 0.10, as opposed to 0.05, was applied for the FDR correction in recognition of the large number of metabolites being tested and our goal of identifying metabolites that would warrant future study as potential biomarkers.

## Results

### Physicochemical Parameters

Measured water parameters were similar within and among treatment levels. Water temperature in the microcosms averaged 22.5°C (range 21.9–22.8°C). Dissolved oxygen was an average of 101.2% (88.4–119.2%). Temperature and dissolved oxygen values did not exhibit any observable trends throughout the duration of the experiment. Specific conductivity was an average of 450.2 μS cm^−1^ (360.1–546.0 μS cm^−1^). Specific conductivity generally increased with time for all treatment levels. pH averaged 8.17 (7.92–8.37) and decreased between Day 0 and Day 7 in treatment levels, but did not exhibit any trends in the following two weeks. Overall, there were no trends in water chemistry that distinguished any treatment from the control.

### *Hexagenia* Survival and Growth

Individual mayflies weighed an average of 15.92 ± 4.31 mg wet weight at the beginning of the experiment. Throughout the duration of the exposure, each mayfly gained an average of 1.09 mg per day, resulting in an average mass of 38.79 ± 6.08 mg at the end of the experiment. Mean mass gained per individual did not vary with treatment level (*p* = 0.892, *F* = 0.019, *R* ^2^ = 0.001).

Survival of mayflies was high among all treatment levels with only 17 of 1,080 individuals across all treatment levels and replicates dying over the duration of the study. Mortality was greatest at the highest treatment level (i.e., 100 μg L^−1^) at 4 individuals for the 4 replicates combined. In contrast, only one individual died at the control level and no more than two died at the other levels of exposure resulting in a combined range of survival from 94.4 to 100%. Three individuals are presumed to have been miscounted at the beginning of the experiment, as one microcosm at the 0.006 μg L^−1^ level had one extra individual and two extra individuals were present at the 0.04 μg L^−1^ level at the conclusion of the experiment. Survival of mayfly nymphs was negatively related with sodium naphthenate concentration (*F* = 3.26, *p* = 0.003, *R* ^2^ = 0.505).

### Polar Metabolome

**FIGURE 2:**
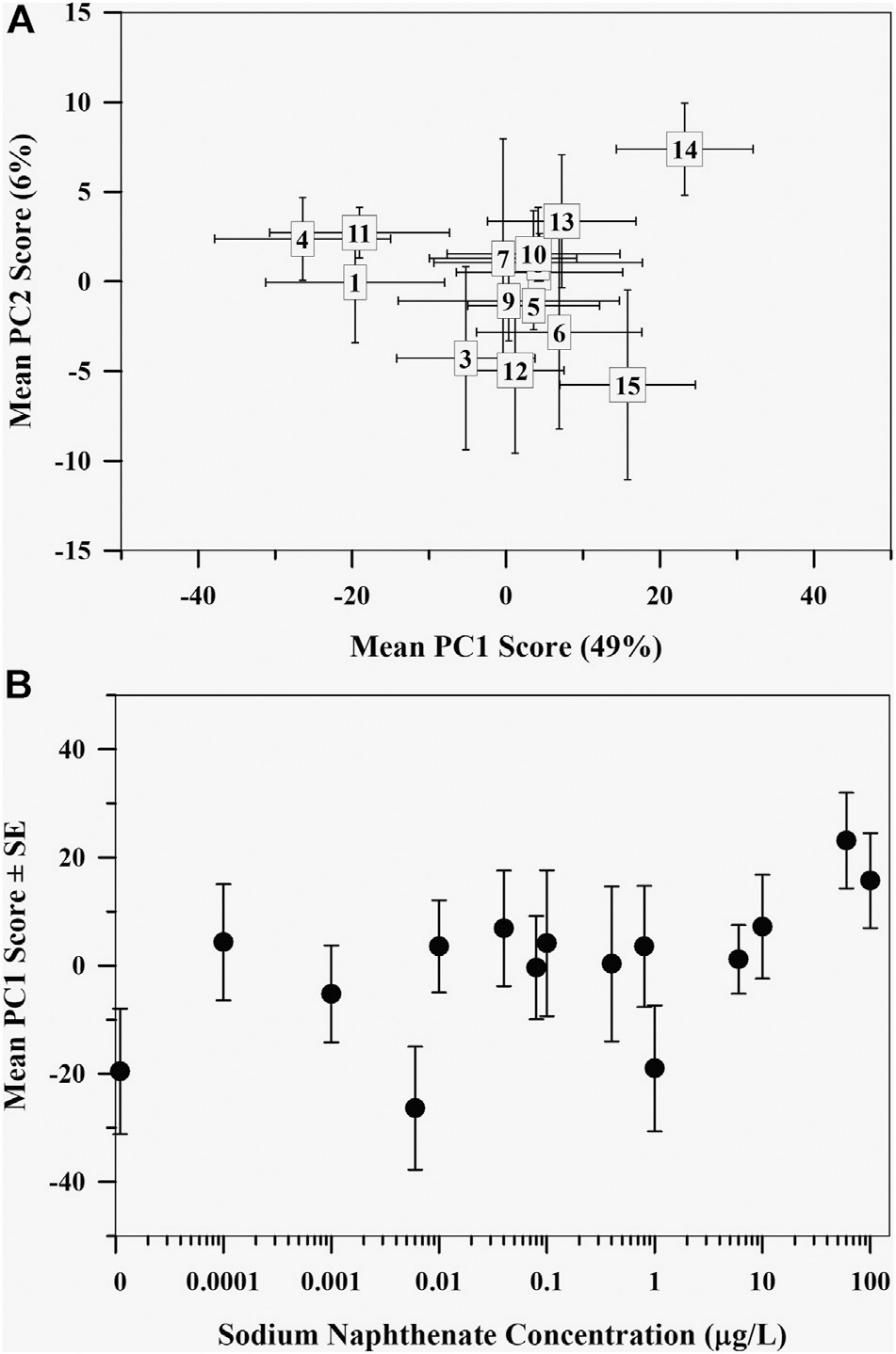
**(A)** Mean principal component scores of the mayfly (*Hexagenia* spp.) nymph metabolome exposed to sodium naphthenate. Numbers represent exposure from lowest 0 μg L^−1^ (1) to highest 100 μg L^−1^ (15) sodium naphthenate concentrations. Error bars indicate ± one standard error of the mean. **(B)** Mean first principal component scores of the mayfly (*Hexagenia* spp.) nymph polar metabolome exposed to sodium naphthenate concentrations (0, 0.0001, 0.001, 0.006, 0.01, 0.04, 0.08, 0.1, 0.4, 0.8, 1, 6, 10, 60, and 100 μg L^−1^). Error bars indicate ± one standard error of the mean. Note the logarithmic scale in concentration along the *x* axis.

The first two axes of the PCA cumulatively explained 54.8% of variation in the polar metabolome data (Figure 2A). There was little additional variance explained by PC3 and higher (≤2.7%). Most separation in the data occurred along PC1, with the control metabolome and the two metabolomes exposed to the highest sodium naphthenate concentrations on opposite ends of PC1 (Figure 2A). Regression analysis revealed a positive association between the mayfly metabolome, represented by the mean PC1 score value, and sodium naphthenate concentration (*F* = 6.34, *p* = 0.026, *R* ^2^ = 0.33) (Figure 2B). No relationship was found between PC2 scores and sodium naphthenate concentrations (*F* = 0.10, *p* = 0.75, *R* ^2^ = 0.001).

PatternHunter analysis of the metabolites found 11 metabolites that correlated with the sodium naphthenate concentrations (Table 1). Arginine, glutamine, aspartate, anserine, creatine, malonate, tyrosine, 2-hydroxybutyrate, and formate decreased with sodium naphthenate concentration. In contrast, adenosine monophosphate (AMP) and inosine monophosphate (IMP) were positively associated with sodium naphthenate concentration.

**TABLE 1:** Metabolites that correlate with increasing naphthenic acid concentrations identified from the PatternHunter analysis of the polar metabolome of the mayfly, *Hexagenia* spp. Chemical shift and multiplicities are reported from Fan (1996) and Chenomx NMR Suite 8.3.

Metabolite	Identified bin (ppm)	Pearson r correlation coefficient	False discovery rate	Chemical shift [ppm, (multiplicity)]
Formate	8.4325	−0.28585	0.063696	**8.46(s)**
Anserine	7.1225	−0.25292	0.070336	2.65(m), 2.71(m), 3.04(dd), 3.20(m), 3.22(dd), 3.23(m), 3.77(s), 4.48(m), **7.12(s)**, 8.22(s), 8.28(d)
AMP	6.1025	0.27444	0.063696	4.01(dd), 4.46(dd), 4.35(dd), **6.04(d)**, 8.12(s), 8.55(s)
IMP	4.4925	0.28918	0.063696	4.01(m), 4.04(m), 4.36(m), **4.50(dd)**, 6.15(d), 8.23(s), 8.57(s)
Tyrosine	3.1575	−0.29089	0.063696	3.05(dd), **3.15(dd)**, 3.93(dd), 6.88(d), 7.18(d)
Malonate	3.1325	−0.28884	0.063696	**3.13(s)**
Creatine	3.0125	−0.27923	0.063696	**3.04(s)**, 3.94(s)
Aspartate	2.8175	−0.34607	0.063696	2.68(dd), **2.79(dd),** 3.89 (dd)
Glutamine	2.4725	−0.27914	0.063696	2.11(m), 2.14(m), 2.43(m), **2.45(m)**, 3.77(t), 6.87(s), 7.59(s)
Arginine	1.8775	−0.26858	0.063696	1.68(m), **1.92(m),** 3.23(t), 3.78(t)
2-Hydroxybutyrate	0.8425	−0.25659	0.068411	**0.85(t),** 1.64(m), 1.73(m), 3.99(dd)

Bolded ppm values in the last column represent the peak which was related to the bin showing significance. Multiplicity identifiers are taken from Fan (1996) and correspond to (s):singlet; (d):doublet; (dd):doublet of doublets; (t):triplet; (dt):doublet of triplets; (q):quartet; and (m):multiplet.

### Lipid Metabolome

**FIGURE 3:**
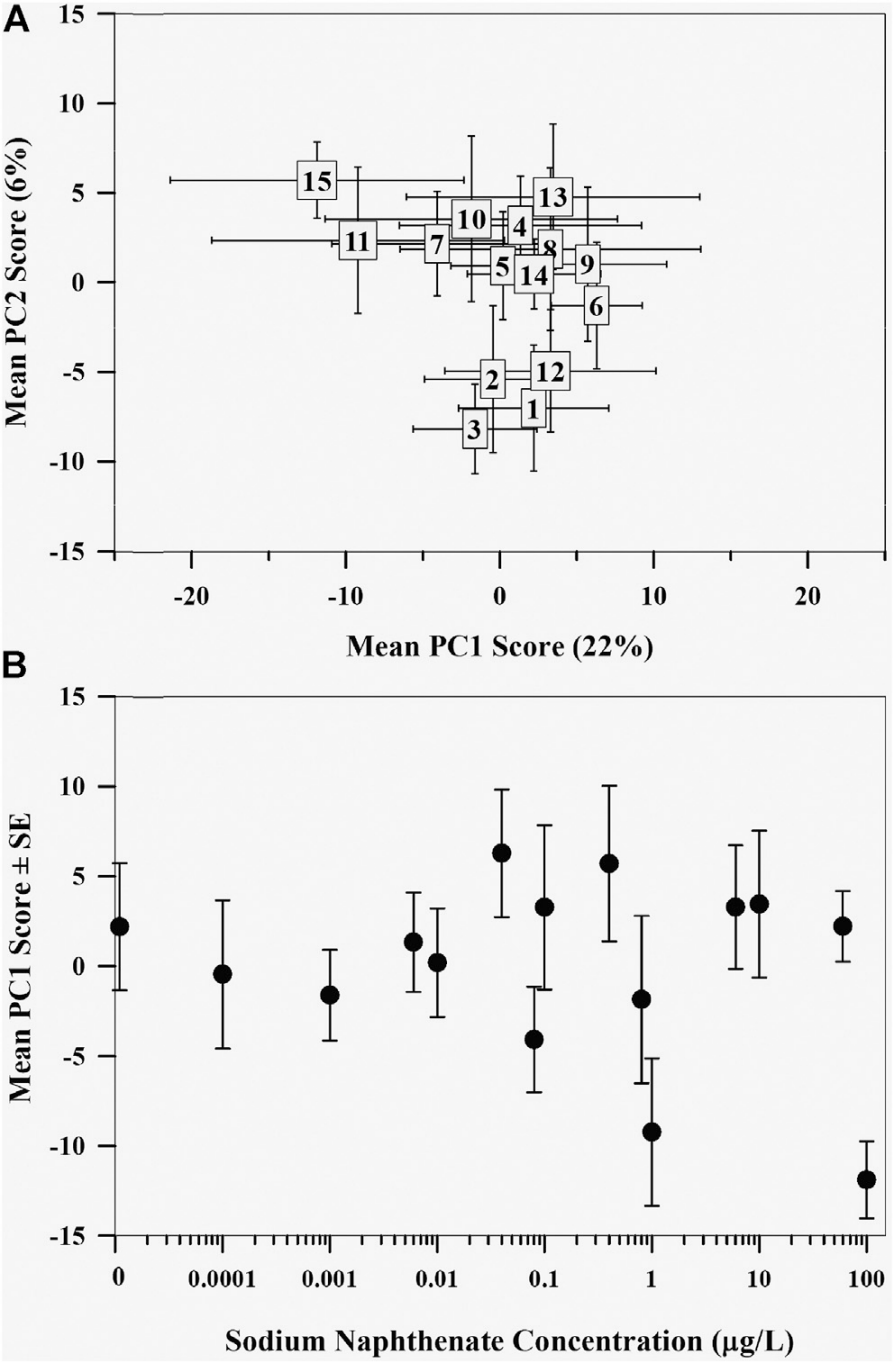
**(A)** Mean principal component scores of the lipid metabolome of mayflies (*Hexagenia* spp.) exposed to sodium naphthenate. Numbers represent exposure from lowest 0 μg L^−1^ (1) to highest 100 μg L^−1^ (15) sodium naphthenate concentrations. Error bars indicate ± one standard error of the mean. **(B)** Mean first principal component scores of the mayfly (*Hexagenia* spp.) nymph lipid metabolome exposed to sodium naphthenate concentrations (0, 0.0001, 0.001, 0.006, 0.01, 0.04, 0.08, 0.1, 0.4, 0.8, 1, 6, 10, 60, and 100 μg L^−1^). Error bars indicate ± one standard error of the mean. Note the logarithmic scale in concentration along the x axis.

The first two principal components explained 28.6% of the total variation, with PC1 explaining 22.2% and PC2 explaining 6.4% (Figure 3A), with little variance explained with additional PC axes (≤4.8%). Although most of the variation explained was along PC1, the control mayfly metabolome and the metabolomes exposed to the highest sodium naphthenate concentration were on opposite ends of PC2. Regression analysis revealed a negative relationship between the mean PC1 score of the mayfly metabolome and sodium naphthenate concentration (*F* = 3.95, *p* = 0.068, *R* ^2^ = 0.17; Figure 3B). No relationship between sodium naphthenate concentration and mean PC2 scores was found (*F* = 1.94, *p* = 0.19, *R* ^2^ = 0.06).

## Discussion

Our investigation revealed that the polar metabolomes of *Hexagenia* mayflies were associated with concentrations of naphthenic acids currently observed in aquatic environments in the surface mining region of the Alberta oil sands, suggesting that increasing exposure, even across the lower concentrations currently observed in rivers and streams in the Alberta oil sands surface mining region, may initiate a stress response. Indeed, mayflies exposed to highest concentrations of naphthenic acids had altered homeostasis with disrupted energy metabolism. Although mayfly growth was not related to naphthenic acid concentrations, mayfly survival was negatively associated with exposure concentration. The presence of physiological effects at currently observed concentrations of naphthenic acids has implications for management of OSPW, as seepage from tailings ponds, accidental release, and the potential future intentional release of treated OSPW to the Athabasca River may have the potential to result in ecosystem scale impacts.

Identified metabolites that correlated with the increasing naphthenic acid concentration in the polar metabolome spanned several biological processes including oxidative phosphorylation, energy metabolism, and the urea cycle, suggesting a breadth of potential adverse exposure outcomes. Previous studies examining the effects of naphthenic acids identified impacts to energy metabolism pathways, including Melvin et al. (2013) who observed a significant decrease in glycogen stores in tadpoles exposed to 1,000 μg L^−1^ naphthenic acid. Our results also indicate alteration of energy metabolism with increasing naphthenic acid exposure as several metabolites related to energy pathways (e.g., AMP, IMP, 2-hydroxybutyrate, aspartate and formate) were associated with the tested exposure gradient. Moreover, our findings are consistent with past studies identifying naphthenic acid induced oxidative stress and suppression of apoptosis (Xu et al., 1997; Matés et al., 2002), as well as changes in gene expression regulating apoptosis in fathead minnows (He et al., 2012) as a consequence of declines in glutamine. In addition, our finding of a negative relationship between naphthenic acid exposure and arginine provides further indication of adverse outcomes of naphthenic acids as decreases in arginine have been widely linked to oxidative stress (Chaitanya et al., 2016) as well as immune responses in aquatic invertebrates (Yina et al., 2016).

Although our study identified similar linkages to adverse outcomes observed in previous studies, it is noteworthy that our study was able to detect similar biochemical responses despite the tested naphthenic acid concentrations in our study being well below those of previous studies. Our findings thus demonstrate the sensitivity of the metabolome as a biological assessment tool even in low exposure situations. Overall, our study adds to a growing body of literature demonstrating the likely initiation of adverse outcome pathways of naphthenic acids and indicates the potential of the metabolome and individual metabolites to serve as effective bioindicators of naphthenic acid stress. Moreover, the identified metabolites can provide a starting point for identifying biomarkers of exposure that can be used in future assays and ultimately for diagnostic field assessments, thereby able to aid in the prediction of higher-level ecosystem impacts and ultimately better inform environmental management decisions.

We had anticipated that the lipid metabolome may be particularly sensitive to naphthenic acids as these compounds have been hypothesized to cause membrane disruption due to surfactant properties (Frank et al., 2009). However, examination of the linear trend suggests that the observed linear association is largely driven by the metabolomes of mayflies exposed to two treatment concentrations orders of magnitude apart. Consequently, it is difficult to draw clear conclusions regarding the sensitivity of the lipid metabolome, and the physiological processes the lipid metabolome represents, to naphthenic acids. It does appear though that existing environmental concentrations (i.e., <60 μg L^−1^) may be insufficient to initiate a response in the lipid metabolome. However, shifts in the lipid metabolome observed at our highest naphthenic acid exposure level indicate that a biochemical response may be initiated at levels approximating our highest treatment level (100 μg L^−1^) suggesting a potential threshold for naphthenic acid effects. Such threshold responses have been observed in other studies assessing non-lethal levels of contaminants using metabolomics (e.g., (Lankadurai et al., 2011a; Lankadurai et al., 2011b; Dani et al., 2018). However, because we limited our study to concentrations currently observed in aquatic environments and the evidence for a potential threshold occurs at the highest concentration tested in our study we cannot confirm this form of response from our results. Experiments testing concentrations greater than those included in our study are therefore needed to more fully discern the response of the lipid metabolome to naphthenic acid exposure.

Despite the observed association between the metabolome and survival of *Hexagenia* exposed to sublethal naphthenic acid concentrations, a corresponding association in growth was not observed. However, exposure effects on sub-organism level indicators, such as the metabolome, coupled with no variation in growth rates has also been observed in other studies. For example, Leclair et al. (2013) found increases in liver size relative to body mass in rainbow trout after exposure to 1,000 μg L^−1^ naphthenic acid for 7 days. Likewise, Howland et al. (2019b) found changes in several morphometrics of *Hexagenia* spp. exposed to varying concentrations of naphthenic acids without a concurrent change in body mass. These findings thus suggest that morphological changes may be precursory to mass changes in multiple species, indicating that a lack of changes in mass does not preclude observable physiological impacts. Thus, while it is possible that due to the nature of the physiological changes, exposure levels of 100 μg L^−1^ or lower may not have a biological consequence it appears from our findings and those of others that these low levels may be increasing organism susceptibility to additional stress.

Our observation of an incremental metabolomic response in the absence of an observed growth effect suggests a possible lag between when effects are visible in the metabolome vs. a result in observable ecological consequences. Such lag effects have been observed in other aquatic taxa that have been exposed to naphthenic acids. Indeed, Philibert et al. (2019) identified transgenerational and behavioral effects in zebrafish exposed to ozonated OSPW containing 600 μg L^−1^ naphthenic acid. If present, the lag between a metabolomic response and an observable fitness response would allow for the metabolome to serve as an early warning of ecologically relevant effects before they occur, increasing effectiveness of management actions. Future research is needed to determine if greater environmental naphthenic acid concentrations have additional biological consequences for mayflies and to elucidate the linkage between increased mortality, metabolomic changes, and potential transgenerational outcomes.

## Conclusion

Our findings have two important implications for future management of naphthenic acids in the environment. First, our study focused on discerning the biological effects of naphthenic acids at concentrations known to occur in aquatic ecosystems in and around the bitumen extraction areas of Alberta, Canada and elsewhere. Thus, the observed response of the mayfly *Hexagenia* spp. to the range of concentrations we evaluated, suggests that naphthenic acid concentrations currently observed in the Athabasca River and many of its tributaries in the oil sands surface mining region of Alberta (i.e., ≤60 μg L^−1^, Arens et al., 2017) may pose little threat to the continued maintenance of *Hexagenia* spp. populations in these ecosystems. However, the observed incremental association between the tested concentrations of naphthenic acid and the polar metabolomes of the tested *Hexagenia* spp. that we observed in our study indicates that additional exposure may lead to physiological changes that could result in changes in organism fitness and ultimately population size. Moreover, the observed decrease in survival, albeit modest (i.e., 6% increase in mortality across tested concentration gradient), further suggests that fitness effects may occur should environmental concentrations be increased beyond those currently observed in the region. As such, future studies need to be conducted to evaluate possible management scenarios that could lead to release of OSPW to aquatic ecosystems and thereby increase the intensity of naphthenic acid exposure to aquatic organisms.

Second, our study demonstrates the potential of the metabolome of *Hexagenia* spp. to serve as a tool for bioassays aimed at identifying non-lethal effects of naphthenic acids that could be used to provide early warning of potential ecosystem level changes resulting from increased naphthenic acid concentrations. Moreover, our study has provided individual metabolites that should be explored in future studies for potential use as diagnostic biomonitoring tools of exposure to naphthenic acids. Together, these metabolomic biomonitoring tools could greatly enhance the effectiveness of aquatic monitoring in regions where naphthenic acids are a stressor of concern and thereby allow more informed management decisions regarding naphthenic acid containing wastes such as OSPW.

## Data Availability

The raw data supporting the conclusions of this article will be made available by the authors, without undue reservation.
